# Determinants of early initiation of breastfeeding among mothers of children aged 0–24 months in Mogadishu, Somalia: a community-based cross-sectional study

**DOI:** 10.1016/j.pmedr.2026.103686

**Published:** 2026-10-27

**Authors:** Ahmed Saed Hussein, Farhiya Abduqadir Ahmed, Samiro Osman Yusuf, Siham Mohamed Dahir, Safa Hashi Adani, Asli Ibrahim Abdrashid, Suad Abdullahi Abdule, Naimo Hirsi Abdi jimale

**Affiliations:** a Faculty of Medicine and Health Sciences, Jamhuriya University of Science and Technology, Mogadishu, Somalia

**Keywords:** Early initiation of breastfeeding, Breastfeeding, Prelacteal feeding, Colostrum, Antenatal care, Caesarean delivery, Somalia, Mogadishu

## Abstract

**Objective:**

To determine the prevalence of early initiation of breastfeeding (EIBF) and its associated factors among mothers of children aged 0–24 months in Mogadishu, Somalia.

**Methods:**

A community-based cross-sectional study was conducted in six selected districts of Mogadishu from March to June 2026. Structured interviews were completed with 638 mothers. Analysis included 561 mothers who had ever breastfed. Multivariable logistic regression identified factors associated with EIBF.

**Results:**

EIBF prevalence was 82.7%. Lower odds were observed among mothers without formal education (adjusted odds ratio [AOR] 0.30, 95% confidence interval [CI] 0.16, 0.53), those attending 2–3 antenatal care visits (AOR 0.17, 95% CI 0.06, 0.46) or four or more visits (AOR 0.09, 95% CI 0.03, 0.27), those without breastfeeding counselling (AOR 0.28, 95% CI 0.15, 0.53), and those delivering by caesarean section (AOR 0.13, 95% CI 0.07, 0.25). Household size of eight or more members and absence of prelacteal feeding were associated with higher odds.

**Conclusions:**

Although EIBF was common, 17.3% initiated breastfeeding after one hour. Strengthening breastfeeding counselling, post-caesarean support and efforts to prevent prelacteal feeding may improve timely initiation.

## Highlights

• Breastfeeding began within one hour for 82.7% of mothers who had breastfed.
• Mothers without formal education had lower odds of timely breastfeeding initiation.
• Caesarean delivery was associated with lower odds of timely breastfeeding initiation.
• Not providing prelacteal feeds was associated with higher odds of timely initiation.
• Findings support counselling and post-caesarean breastfeeding support in Mogadishu.

## 1 Introduction

Early initiation of breastfeeding (EIBF), defined as putting the newborn to the breast within one hour of birth, is an important intervention for improving newborn health and survival. Timely initiation enables newborns to receive colostrum, which contains immunoglobulins, antimicrobial components and other bioactive substances that support early immune protection (Takahashi et al., 2017; Victora et al., 2016). Supportive maternity-care practices promoted through the Baby-friendly Hospital Initiative can improve breastfeeding outcomes (Pérez-Escamilla et al., 2016). Nevertheless, delayed initiation remains common in many low- and middle-income countries.

Globally, EIBF prevalence varies considerably. An analysis of the World Health Organization Global Survey across 24 countries reported a mean prevalence of 57.6% (Takahashi et al., 2017). Studies in sub-Saharan Africa have identified maternal characteristics, residence, antenatal care (ANC), delivery care and socioeconomic factors as correlates of EIBF (Birhan et al., 2022; Nkoka et al., 2019; Terefe et al., 2024; Seidu et al., 2020). Research in Ethiopia, Nigeria, The Gambia and other settings has also highlighted maternal education, ANC utilisation, place and mode of delivery, and health-service factors (Abebe et al., 2024; Ayalew et al., 2022; Berde and Yalcin, 2016; Darboe et al., 2023; Liben and Yesuf, 2016; Woldeamanuel, 2020). Evidence from Asia similarly demonstrates variation according to maternal, household, obstetric and health-service characteristics (Senanayake et al., 2019; Sharma and Byrne, 2016; Hernández-Vásquez and Chacón-Torrico, 2019).

Breastfeeding counselling and supportive maternity care may facilitate timely initiation, whereas caesarean delivery can create barriers through postoperative pain, anaesthesia, maternal immobility and delayed mother–infant contact (Pérez-Escamilla et al., 2016; Alrasheedi, 2023; Sako et al., 2022). Prelacteal feeding has also been associated with delayed initiation and may reduce opportunities for immediate breastfeeding (Liben and Yesuf, 2016; Nkoka et al., 2019). Cultural beliefs surrounding colostrum may influence breastfeeding practices, although associations vary across settings (Sharma and Byrne, 2016; Horii et al., 2017). These findings indicate that EIBF reflects interacting maternal, household, healthcare and sociocultural factors.

In Somalia, evidence from primary community-based studies examining detailed breastfeeding practices and health-service factors remains limited. An analysis of the 2020 Somali Demographic and Health Survey reported that 75% of mothers initiated breastfeeding within one hour of birth and identified maternal age, residence, radio exposure, maternal employment, husband's employment and facility delivery as associated factors (Ahmed et al., 2026). However, national survey data provide limited information on breastfeeding counselling during ANC, prelacteal feeding and colostrum practices.

Community-based evidence from the city can complement national survey findings and help identify context-specific opportunities to improve breastfeeding practices. Therefore, this study aimed to determine the prevalence of EIBF and identify factors associated with the practice among mothers of children aged 0–24 months in selected districts of Mogadishu, Somalia.

## 2 Methods

### 2.1 Study design and population

A community-based cross-sectional study was conducted among mothers of children aged 0–24 months in six selected districts of Mogadishu, Somalia: Dharkenley, Deynile, Hodan, Wadajir, Kaxda and Garasbaaley. The study population comprised mothers with biological children aged 0–24 months residing in the selected districts during the study period. Data collection took place from March to June 2026.

The sample size was calculated using the single population proportion formula, assuming 50% prevalence, 95% confidence level and 5% margin of error, yielding an initial sample of 384. A design effect of 1.5 increased the sample to 576; allowing for 10% non-response resulted in a target of 640 mothers. A total of 638 mothers completed the interview (response rate, 99.7%).

A multistage sampling procedure was used. First, six of Mogadishu's 20 administrative districts were selected by lottery. The number of districts included reflected practical considerations, including the available budget, time and fieldwork capacity. Within the selected districts, data collectors visited households and recruited eligible mothers who were available and willing to participate until the allocated sample size was reached. Because a complete and reliable household sampling frame was unavailable, convenience sampling was used for participant recruitment at this stage. When more than one eligible mother was present in a household, one was selected by lottery. If a mother had more than one child aged 0–24 months, the youngest eligible child was selected to minimise recall bias. The approach was adapted from Liben and Yesuf (2016).

### 2.2 Measures

Data were collected through face-to-face interviews using a structured, interviewer-administered questionnaire covering sociodemographic characteristics, maternal and health-service utilisation, obstetric characteristics and breastfeeding practices. Variables included maternal age, education, marital status, household size, parity, antenatal care (ANC) utilisation, breastfeeding counselling during pregnancy, place and mode of delivery, delivery attendant, postnatal care, colostrum practices and prelacteal feeding.

The primary outcome was early initiation of breastfeeding (EIBF), defined as initiation within one hour after birth, consistent with the World Health Organization recommendation. Mothers reported the interval between delivery and the first breastfeeding attempt. EIBF was coded as 1 for initiation within one hour and 0 for initiation after one hour. The analysis was restricted to mothers who had ever breastfed the index child (*n* = 561).

### 2.3 Statistical analysis

Data were checked for completeness and coded before statistical analysis. Categorical variables were summarised using frequencies and percentages. Bivariable logistic regression examined associations between explanatory variables and early initiation of breastfeeding (EIBF). Variables with *p*-values below 0.25 in bivariable analyses were considered for inclusion in the multivariable logistic regression model. Results were reported as crude odds ratios (CORs) and adjusted odds ratios (AORs), with 95% confidence intervals (CIs). Statistical significance was defined as a two-sided *p*-value below 0.05. The Hosmer–Lemeshow goodness-of-fit test assessed model fit, and variance inflation factors were examined for multicollinearity. All statistical analyses were performed using Stata version 19 (StataCorp, College Station, TX, USA).

### 2.4 Ethical considerations

Ethical approval was obtained from the Jamhuriya University of Science and Technology Research Ethics Committee (JUREC), Mogadishu, Somalia (approval reference: JUREC0205/FMHS 419/2026). Written informed consent was obtained from all participants after they had received information about the study objectives and procedures. Participation was voluntary, and participants were informed of their right to withdraw at any time without consequences. Confidentiality and anonymity were maintained throughout data collection, analysis, and reporting.

## 3 Results

A total of 638 mothers of children aged 0–24 months participated in the study (response rate, 99.7%). Most mothers were aged 25–34 years (53.1%), while 41.5% were aged 15–24 years and 5.3% were aged 35 years or older. Formal education had been received by 58.6% of mothers and 67.7% of fathers. Most participants were married (84.6%), and the father was the household head in 78.8% of households. Nearly half of households (49.2%) had one to four members (Table 1).

**Table 1:** Sociodemographic characteristics of mothers of children aged 0–24 months in selected districts of Mogadishu, Somalia (*N* = 638) during March–June 2026.

Characteristic	Category	n (%)
Maternal age (years)	15–24	265 (41.5)
	25–34	339 (53.1)
	35 or older	34 (5.3)
Maternal educational status	Educated	374 (58.6)
	Uneducated	264 (41.4)
Marital status	Married	540 (84.6)
	Divorced	79 (12.4)
	Widowed	19 (3.0)
Household head	Father	503 (78.8)
	Mother	135 (21.2)
Paternal educational status	Educated	432 (67.7)
	Uneducated	206 (32.3)
Household size	1–4 members	314 (49.2)
	5–7 members	187 (29.3)
	8 or more members	137 (21.5)

Note: Values are numbers and percentages of participating mothers (*N* = 638). Percentages may not total 100 because of rounding.

Most mothers were multiparous (80.4%), and 40.8% reported that the index child was their second to fourth birth. Nearly half (49.1%) attended 2–3 antenatal care (ANC) visits, while 25.7% attended four or more visits. Breastfeeding counselling during ANC was reported by 64.3% of mothers. Most delivered in a health institution (82.1%), were assisted by a health professional (85.6%) and had a vaginal delivery (86.1%). Postnatal care was utilised by 79.8% (Table 2).

**Table 2:** Maternal, obstetric and health-service characteristics of mothers of children aged 0–24 months in selected districts of Mogadishu, Somalia (*N* = 638) during March–June 2026.

Characteristic	Category	n (%)
Parity	Primipara	125 (19.6)
	Multipara	513 (80.4)
Birth order	First child	194 (30.4)
	Second to fourth	260 (40.8)
	Fifth or higher	184 (28.8)
Antenatal care visits	0–1 visit	161 (25.2)
	2–3 visits	313 (49.1)
	4 or more visits	164 (25.7)
Received breastfeeding counselling during antenatal care	Yes	410 (64.3)
	No	228 (35.7)
Place of delivery	Health institution	524 (82.1)
	Home	114 (17.9)
Delivery attendant	Health professional	546 (85.6)
	Traditional birth attendant	92 (14.4)
Mode of delivery	Vaginal	549 (86.1)
	Caesarean section	89 (13.9)
Postnatal care utilisation	Yes	509 (79.8)
	No	129 (20.2)

Note: Values are numbers and percentages of participating mothers (*N* = 638). Percentages may not total 100 because of rounding.

Of the 638 mothers, 561 (87.9%) had ever breastfed and were included in the breastfeeding-practice and early initiation of breastfeeding (EIBF) analyses. Among these mothers, 464 (82.7%) initiated breastfeeding within one hour of birth, whereas 97 (17.3%) initiated breastfeeding later. Colostrum avoidance was reported by 45.1%, while 54.9% did not avoid colostrum. Similarly, 54.9% reported prelacteal feeding, while 45.1% did not (Table 3).

**Table 3:** Breastfeeding practices among mothers of children aged 0–24 months in selected districts of Mogadishu, Somalia during March–June 2026.

Breastfeeding practice	Category	n (%)
Ever breastfed (*N* = 638)	Yes	561 (87.9)
	No	77 (12.1)
Early initiation of breastfeeding (*n* = 561)	Within 1 h after birth	464 (82.7)
	More than 1 h after birth	97 (17.3)
Colostrum avoidance (n = 561)	Yes	253 (45.1)
	No	308 (54.9)
Prelacteal feeding (n = 561)	Yes	308 (54.9)
	No	253 (45.1)

Note: Values are numbers and percentages. The total sample was 638 mothers; analyses of early initiation of breastfeeding, colostrum avoidance and prelacteal feeding were restricted to the 561 mothers who had ever breastfed the index child.

In bivariable analyses, several variables were associated with EIBF and were considered for inclusion in the multivariable model. After adjustment, maternal education, household size, ANC utilisation, breastfeeding counselling during ANC, mode of delivery, colostrum avoidance and prelacteal feeding remained independently associated with EIBF (Table 4).

**Table 4:** Factors associated with early initiation of breastfeeding among mothers of children aged 0–24 months in selected districts of Mogadishu, Somalia (n = 561) during March–June 2026.

Variable	Category	Crude odds ratio (95% confidence interval)	Adjusted odds ratio (95% confidence interval)
Maternal age (years)	15–24	1.00 (Reference)	1.00 (Reference)
	25–34	0.58 (0.29, 1.15)	1.29 (0.69, 2.43)
	35 or older	0.32 (0.09, 1.14)	0.84 (0.23, 3.12)
Maternal educational status	Educated	1.00 (Reference)	1.00 (Reference)
	Uneducated	0.30 (0.18, 0.50)	0.30 (0.16, 0.53)
Household size	1–4 members	1.00 (Reference)	1.00 (Reference)
	5–7 members	1.67 (0.94, 2.97)	1.82 (0.87, 3.79)
	8 or more members	4.39 (1.77, 10.91)	4.68 (1.72, 12.70)
Antenatal care visits	0–1	1.00 (Reference)	1.00 (Reference)
	2–3	0.39 (0.17, 0.86)	0.17 (0.06, 0.46)
	4 or more	0.25 (0.11, 0.58)	0.09 (0.03, 0.27)
Breastfeeding counselling during antenatal care	Yes	1.00 (Reference)	1.00 (Reference)
	No	0.50 (0.31, 0.81)	0.28 (0.15, 0.53)
Mode of delivery	Vaginal delivery	1.00 (Reference)	1.00 (Reference)
	Caesarean section	0.13 (0.08, 0.22)	0.13 (0.07, 0.25)
Colostrum avoidance	Yes	1.00 (Reference)	1.00 (Reference)
	No	0.34 (0.21, 0.55)	0.40 (0.21, 0.75)
Prelacteal feeding	Yes	1.00 (Reference)	1.00 (Reference)
	No	2.07 (1.29, 3.30)	4.29 (2.29, 8.04)

Note: Values are odds ratios with 95% confidence intervals. “Reference” indicates the comparison category. The adjusted logistic regression model included maternal age, maternal educational status, household size, antenatal care visits, breastfeeding counselling during antenatal care, mode of delivery, colostrum avoidance and prelacteal feeding.

Compared with educated mothers, mothers without formal education had lower odds of EIBF (adjusted odds ratio [AOR] = 0.30, 95% confidence interval [CI]: 0.16, 0.53). Mothers living in households with eight or more members had higher odds of EIBF than those living in households with one to four members (AOR = 4.68, 95% CI: 1.72, 12.70).

Compared with mothers attending 0–1 ANC visits, those attending 2–3 visits (AOR = 0.17, 95% CI: 0.06, 0.46) or four or more visits (AOR = 0.09, 95% CI: 0.03, 0.27) had lower odds of EIBF. Mothers who did not receive breastfeeding counselling during ANC had lower odds of EIBF than those who received counselling (AOR = 0.28, 95% CI: 0.15, 0.53). Caesarean delivery was also associated with lower odds of EIBF than vaginal delivery (AOR = 0.13, 95% CI: 0.07, 0.25).

Compared with mothers who reported discarding colostrum, those who did not discard it had lower odds of EIBF (AOR = 0.40, 95% CI: 0.21, 0.75). Conversely, mothers who did not provide prelacteal feeds had higher odds of EIBF than those who did (AOR = 4.29, 95% CI: 2.29, 8.04).

## 4 Discussion

This community-based cross-sectional study found that 82.7% of mothers who had ever breastfed initiated breastfeeding within one hour of birth. This prevalence was higher than the 75% reported in the 2020 Somali Demographic and Health Survey (DHS) analysis (Ahmed et al., 2026), and the approximately 57.6% reported in the WHO Global Survey and studies from sub-Saharan Africa and South Asia (Takahashi et al., 2017; Birhan et al., 2022; Sharma and Byrne, 2016; Hernández-Vásquez and Chacón-Torrico, 2019). These differences may reflect variations in study populations and settings: the DHS analysis was nationally representative, whereas this study included mothers from selected urban districts.

Maternal education and household size were independently associated with EIBF. Mothers without formal education had lower odds of timely initiation than educated mothers (AOR = 0.30, 95% CI: 0.16, 0.53), consistent with findings from Ethiopia and other low- and middle-income settings (Ayalew et al., 2022; Liben and Yesuf, 2016; Sharma and Byrne, 2016). Education may support maternal health literacy and awareness of breastfeeding recommendations. However, maternal education was not among the principal determinants reported in the national Somali DHS analysis (Ahmed et al., 2026), suggesting that associated factors may differ across populations and settings.

Mothers living in households with eight or more members had higher odds of early initiation of breastfeeding (EIBF) than those living in households with one to four members (adjusted odds ratio [AOR] = 4.68, 95% confidence interval [CI]: 1.72, 12.70). This association may reflect greater availability of practical support from other household members during the immediate postnatal period. However, household support was not directly assessed in this study, so this explanation remains speculative. The observed association may also reflect unmeasured sociocultural or socioeconomic factors or residual confounding and should therefore be interpreted cautiously.

An unexpected inverse association was observed between antenatal care (ANC) utilisation and early initiation of breastfeeding (EIBF). Compared with mothers who attended zero to one ANC visit, those who attended two to three visits and those who attended four or more visits had lower adjusted odds of EIBF (adjusted odds ratio [AOR] = 0.17, 95% confidence interval [CI]: 0.06, 0.46, and AOR = 0.09, 95% CI: 0.03, 0.27, respectively). The questionnaire assessed the number of ANC visits during the pregnancy of the index child, and the recorded visit counts were consistent with the categories used in the analysis. However, the reasons for this inverse association remain unclear. The number of ANC visits alone does not indicate whether mothers received breastfeeding counselling or reflect the content and quality of care provided. Residual confounding and unmeasured differences in maternal characteristics or healthcare experiences may also have contributed to the observed association. This finding should not be interpreted as evidence that greater ANC attendance reduces EIBF. Further research is needed to investigate this relationship in Somalia.

In contrast, mothers who did not receive breastfeeding counselling during ANC had lower odds of EIBF than those who received counselling (AOR = 0.28, 95% CI: 0.15, 0.53). Caesarean delivery was also associated with lower odds of EIBF (AOR = 0.13, 95% CI: 0.07, 0.25), consistent with findings from Ethiopia, Saudi Arabia and other settings (Ayalew et al., 2022; Alrasheedi, 2023; Sako et al., 2022). Postoperative pain, anaesthesia, maternal immobility and delayed mother–infant contact may impede timely initiation. Mothers who did not provide prelacteal feeds had higher odds of EIBF than those who did (AOR = 4.29, 95% CI: 2.29, 8.04), consistent with evidence from Ethiopia, Bangladesh and other low- and middle-income settings (Liben and Yesuf, 2016; Sako et al., 2022). Prelacteal feeding may delay breastfeeding and reduce opportunities for immediate breast milk and colostrum intake. Addressing this practice may therefore support timely initiation.

The association between colostrum practices and EIBF was unexpected. Mothers who did not discard colostrum had lower odds of EIBF than those who reported discarding it (AOR = 0.40, 95% CI: 0.21, 0.75). This finding contrasts with the established benefits of colostrum and previous evidence linking colostrum avoidance with suboptimal breastfeeding practices (Victora et al., 2016; Ahmed et al., 2019). The reasons for this association remain uncertain. Differences in the interpretation of the survey question, the relationship between colostrum practices and prelacteal feeding, local cultural practices or residual confounding may have contributed. The finding should not be interpreted as evidence that discarding colostrum is beneficial.

This study had several strengths, including its community-based design, high response rate (99.7%) and assessment of sociodemographic, obstetric, health-service and newborn-feeding factors. Primary data collection also enabled assessment of breastfeeding counselling, prelacteal feeding and colostrum practices that were not adequately captured in the national DHS analysis (Ahmed et al., 2026). However, convenience sampling during final participant recruitment may have introduced selection bias and limits generalisability beyond the selected districts. The cross-sectional design precludes causal inference, while self-reported breastfeeding initiation may be affected by recall and social desirability bias, particularly for children aged up to 24 months. Residual confounding from maternal breastfeeding knowledge, cultural beliefs, family support, skin-to-skin contact, maternal or newborn complications and facility-level breastfeeding practices is also possible.

## 5 Conclusions

EIBF was common among mothers in selected districts of Mogadishu, but 17.3% initiated breastfeeding after the recommended first hour. Maternal education, household size, ANC utilisation, breastfeeding counselling, mode of delivery, colostrum practices and prelacteal feeding were independently associated with EIBF. Strengthening breastfeeding counselling, providing timely support after caesarean delivery and addressing prelacteal feeding may help improve breastfeeding initiation. The findings should be interpreted in light of the cross-sectional design and the use of non-probability sampling during final participant recruitment.

## CRediT authorship contribution statement

**Ahmed Saed Hussein:** Writing – original draft, Supervision, Project administration, Methodology, Investigation, Formal analysis, Data curation, Conceptualization. **Farhiya Abduqadir Ahmed:** Investigation. **Samiro Osman Yusuf:** Investigation. **Siham Mohamed Dahir:** Investigation. **Safa Hashi Adani:** Investigation. **Asli Ibrahim Abdrashid:** Investigation. **Suad Abdullahi Abdule:** Investigation. **Naimo Hirsi Abdi jimale:** Investigation.

## Consent for publication

Not applicable. No individual-level identifiable information or identifiable images are presented in this manuscript.

## Funding

This study received no specific grant from any funding agency in the public, commercial or not-for-profit sectors.

## Declaration of competing interest

The authors declare that they have no known competing financial interests or personal relationships that could have appeared to influence the work reported in this paper.

## Data Availability

The datasets generated and/or analysed during the current study are not publicly available because of ethical and confidentiality considerations but may be available from the corresponding author upon reasonable request and subject to appropriate ethical approval.

## References

[bb0005] Abebe G.F., Tilahun M., Tadesse H., Seid A., Yigremachew T., Birhanu A.M. (2024). Predictors of delayed initiation of breast milk and exclusive breastfeeding in Ethiopia: a multi-level mixed-effect analysis. PLoS One.

[bb0010] Ahmed K.Y., Page A., Arora A., Ogbo F.A. (2019). Trends and determinants of early initiation of breastfeeding and exclusive breastfeeding in Ethiopia from 2000 to 2016. Int. Breastfeed. J..

[bb0015] Ahmed N.R., Dirie A.M., Dirie S.A., Hussein A.S., Hassan Y.S.A., Jimale K.A. (2026). Determinants of early initiation of breastfeeding in Somalia: a population based study using the 2020 demographic and health survey data. J. Neonatal-Perinatal Med..

[bb0020] Alrasheedi A.T. (2023). Factors associated with early initiation of breastfeeding in Central Saudi Arabia: a hospital-based survey. Int. Breastfeed. J..

[bb0025] Ayalew D.D., Kassie B.A., Hunegnaw M.T., Gelaye K.A., Belew A.K. (2022). Determinants of early initiation of breastfeeding in west Belessa District, Northwest Ethiopia. Nutr. Metab. Insights.

[bb0030] Berde A.S., Yalcin S.S. (2016). Determinants of early initiation of breastfeeding in Nigeria: a population-based study using the 2013 demograhic and health survey data. BMC Pregnancy Childbirth.

[bb0035] Birhan T.Y., Alene M., Seretew W.S., Taddese A.A. (2022). Magnitude and determinants of breastfeeding initiation within one hour among reproductive women in sub-Saharan Africa; evidence from demographic and health survey data: a multilevel study. BMC Public Health.

[bb0040] Darboe M.L., Jeyakumar A., Mansour S.M.A., Valawalkar S. (2023). Determinants of early initiation of breastfeeding in the Gambia: a population-based study using the 2019–2020 demographic and health survey data. Int. Breastfeed. J..

[bb0045] Hernández-Vásquez A., Chacón-Torrico H. (2019). Determinants of early initiation of breastfeeding in Peru: analysis of the 2018 demographic and family health survey. Epidemiol. Health.

[bb0050] Horii N., Allman J., Martin-Prével Y., Waltisperger D. (2017). Determinants of early initiation of breastfeeding in rural Niger: cross-sectional study of community based child healthcare promotion. Int. Breastfeed. J..

[bb0055] Liben M.L., Yesuf E.M. (2016). Determinants of early initiation of breastfeeding in Amibara district, northeastern Ethiopia: a community based cross-sectional study. Int. Breastfeed. J..

[bb0060] Nkoka O., Ntenda P.A.M., Kanje V., Milanzi E.B., Arora A. (2019). Determinants of timely initiation of breast milk and exclusive breastfeeding in Malawi: a population-based cross-sectional study. Int. Breastfeed. J..

[bb0065] Pérez-Escamilla R., Martinez J.L., Segura-Pérez S. (2016). Impact of the baby-friendly hospital initiative on breastfeeding and child health outcomes: a systematic review. Matern. Child Nutr..

[bb0070] Sako S., Gilano G., Tekabe B., Abebe S. (2022). Determinants of early initiation of breast feeding among mothers of children aged less than 24 months in Ethiopia: a community-based cross-sectional study. BMJ Open.

[bb0075] Seidu A.-A., Ameyaw E.K., Ahinkorah B.O., Bonsu F. (2020). Determinants of early initiation of breastfeeding in Ghana: a population-based cross-sectional study using the 2014 demographic and health survey data. BMC Pregnancy Childbirth.

[bb0080] Senanayake P., O’Connor E., Ogbo F.A. (2019). National and rural-urban prevalence and determinants of early initiation of breastfeeding in India. BMC Public Health.

[bb0085] Sharma I.K., Byrne A. (2016). Early initiation of breastfeeding: a systematic literature review of factors and barriers in South Asia. Int. Breastfeed. J..

[bb0090] Takahashi K., Ganchimeg T., Ota E., Vogel J.P., Souza J.P., Laopaiboon M. (2017). Prevalence of early initiation of breastfeeding and determinants of delayed initiation of breastfeeding: secondary analysis of the WHO global survey. Sci. Rep..

[bb0095] Terefe B., Belachew T.B., Asmamaw D.B., Wassie G.T., Azene A.G., Eshetu H.B. (2024). Determinants of early initiation of breastfeeding following birth in West Africa: a multilevel analysis using data from multi-country national health surveys. PLoS One.

[bb0100] Victora C.G., Bahl R., Barros A.J.D., França G.V.A., Horton S., Krasevec J. (2016). Breastfeeding in the 21st century: epidemiology, mechanisms, and lifelong effect. Lancet.

[bb0105] Woldeamanuel B.T. (2020). Trends and factors associated to early initiation of breastfeeding, exclusive breastfeeding and duration of breastfeeding in Ethiopia: evidence from the Ethiopia demographic and health survey 2016. Int. Breastfeed. J..

